# A Satisficing Framework for Environmental Policy Under Model Uncertainty

**DOI:** 10.1007/s10666-021-09761-x

**Published:** 2021-03-22

**Authors:** Stergios Athanasoglou, Valentina Bosetti, Laurent Drouet

**Affiliations:** 1 University of Milan - Bicocca, Milan, Italy; 2 Bocconi University, Milan, Italy; 3 CMCC and EIEE, Milan, Italy

**Keywords:** Satisficing, Model uncertainty, Climate change, Computational geometry, C60, D81, Q42, Q48

## Abstract

We propose a novel framework for the economic assessment of environmental policy. Our main point of departure from existing work is the adoption of a *satisficing*, as opposed to optimizing, modeling approach. Along these lines, we place primary emphasis on the extent to which different policies meet a set of goals at a specific future date instead of their performance vis-a-vis some intertemporal objective function. Consistent to the nature of environmental policymaking, our model takes explicit account of model uncertainty. To this end, the decision criterion we propose is an analog of the well-known success-probability criterion adapted to settings characterized by model uncertainty. We apply our criterion to the climate-change context and the probability distributions constructed by Drouet et al. (2015) linking carbon budgets to future consumption. Insights from computational geometry facilitate computations considerably and allow for the efficient application of the model in high-dimensional settings.

## Introduction

Policy makers want direct answers to simple questions, yet such demands are frequently at odds with the complexity of economic analysis and forecasting. The economic assessment of environmental policy, an enterprise often beset by multiple layers of uncertainty, provides a salient case in point.

To fix ideas, consider the setting of climate-change policy. The economics of climate change are characterized by two fundamental challenges. First, there is deep uncertainty regarding the dynamic response of the climate to emissions, the damages higher temperatures will cause to economic activity, and the costs of climate-change mitigation and adaptation. The uncertainty surrounding these crucial modeling inputs falls under the category of *model uncertainty* (Marinacci [1]), meaning that it cannot be captured by unique Bayesian priors. Second, there is strong disagreement regarding the underlying *ethical objective* that policies should strive to meet. These are manifested in vigorous debates regarding the functional form of the objective function, its coefficients of intertemporal substitution and risk aversion, and the magnitude of future discount rates (for a particularly vehement exchange between two eminent economists see Roemer [2, 3] and Dasgupta [4, 5]). Preferences over such parameter values tend to reflect different fundamental ethical stances. As illustrated by the Roemer-Dasgupta conflict, adjudicating between them is a matter of subjective judgment and/or political debate that cannot be resolved via empirical analysis.

Despite these difficulties, the current paper rigorously engages with policy makers’ concerns for clarity and simplicity. It does so by posing the following question, versions of which recur in the global negotiations regarding climate change: *Given the deep uncertainty surrounding environmental decision making, which policy ensures that adverse future impacts are avoided with highest confidence?* To address this question, we adopt a so-called *satisficing*, as opposed to optimizing, modeling framework. First introduced by Herbert Simon in the nineteen-fifties [6–8], satisficing models assume that people reason in terms of meeting a goal (or, alternatively, respecting a constraint), not of optimizing some objective.^1^ Over the years, they have been shown to hold significant descriptive power [9] as well as normative appeal [10–12]. The specific decision-making criterion we propose can be viewed as an analog of the well-known *success-probability* criterion [10, 11] adapted to settings characterized by model uncertainty. The uncertainty sets that form the backbone of our analysis are the *convex hulls* of probability distributions that are relevant to our setting, a choice that is suitable for our practical purposes and often discussed in the theoretical literature (e.g., Ahn [13], Olszewski [14], Danan et al. [15]). We exploit results from computational geometry [16, 17] to propose an efficient method of exactly computing the value of this decision criterion. Under certain assumptions on the constraint set that render it a convex polytope, our geometric technique can accommodate high-dimensional problem domains and multiple goals and indicators. While our focus is on the environmental setting, we emphasize that the decision criterion that we employ, i.e., a success-probability criterion accounting for model uncertainty, is completely general and can be applied to any context of decision-making under model uncertainty.

In the paper’s numerical section, we apply our theoretical model to data by Drouet et al. [18]. Combining comprehensive data from the most recent IPCC AR5 reports with a novel statistical framework, these authors derived a range of plausible probabilistic estimates connecting carbon budgets to climate-change impacts given latest scientific knowledge. These differing estimates correspond to different, but plausible, assumptions regarding mitigation costs, climate dynamics, and climate damages. As such, they reflect the multiplicity of expert opinion on these issues, embodying the model uncertainty alluded to earlier. The main, tentative, result which emerges from our analysis is the superior performance of middle-of-the-road carbon budgets in containing future consumption losses to non-catastrophic levels with high probability. We note that we are wary of drawing policy implications from this numerical exercise; instead, we view its primary function as a proof of concept for the proposed decision-making criterion.

Related work in environmental economics has applied satisficing concepts to dynamic models of sustainable resource management. De Lara and Martinet [19] proposed a stochastic, dynamic-satisficing (referred to also as “stochastic viability”) framework for resource management and computed optimal control rules under an extensive set of monotonicity assumptions on dynamics and constraints. Beyond its adoption of a satisficing as opposed to optimizing framework^2^ , a distinctive feature of their model is its focus on multiple criteria of economic and environmental performance. Martinet [20] and Doyen and Martinet [21] made an explicit connection between stochastic-viability models and sustainability concepts such as the maximin criterion. Doyen et al. [22] and Martinet et al. [23] applied similar ideas to a setting of sustainable fishery management. In the stochastic component of this work, emphasis was placed on calculating the probability of different policies respecting the various sustainability constraints. Where applicable, this was done via Monte Carlo simulation.

A well-known methodology for dealing with model uncertainty in a satisficing framework is Robust Decision Making (RDM), developed by researchers in the RAND Corporation (Lempert et al [24, 25]). RDM proposes a systematic, simulation-based procedure of exploring the implications of many plausible models and synthesizing the resulting information. In particular,“...Rather than using computer models and data as predictive tools, the [RDM] approach runs models myriad times to stress test proposed decisions against a wide range of plausible futures. Analysts then use visualization and statistical analysis of the resulting large database of model runs to help decision-makers identify the key features that distinguish those futures in which their plans meet and miss their goals...” (Lempert [26] page 25). The key goal of RDM is to identify strategies that perform well across a range of plausible modeling assumptions. RDM is a mature methodology that has been applied in many settings, including climate change policy [27].

Our work differs from the previous literature in substantive ways. First, as regards its relation to the stochastic viability literature, our model accounts for model uncertainty by considering multiple probability distributions that link policy choices to future economic and environmental outcomes. This contrasts with [19–23] who incorporate stochasticity but do not take model uncertainty into account. Another important difference in this context involves our work’s focus on one-shot future goals (e.g., sustainability guarantees for the year 2100) as opposed to dynamic constraints in optimal-control settings. Finally, unlike both stochastic viability and RDM, our work does not rely on simulation as a tool for calculating success probabilities, as it exploits the problem’s structure to provide *exact numbers* for these probabilities. Along related lines, the geometric techniques we employ allow us to efficiently study the implications of an (uncountably) infinite set of plausible probability distributions linking current policies to future impacts.

The paper is organized as follows. Section 2 introduces the model and formally defines the decision-making criterion we adopt. Section 3 applies the model to climate-change data by Drouet et al [18]. Section 4 concludes and an Appendix collects all Figures and supplementary analyses.

## Theoretical Model

The theoretical model that is presented in this section is cast in terms of the climate-change application. Thus, model parameters, variables, and probability distributions refer directly to the climate-change context. This is done for the sake of convenience and clarity and results in no loss of generality.

The model’s decision variable is the *carbon budget*, which, for the purposes of the current study, is defined as cumulative CO$_2$ emissions over the period 2010-2100, indexed by *b*. Carbon budgets enjoy favor within the climate-modeling community for their robust statistical relation to global warming [28, 29] and clear translation into policy [30]. We elaborate more on carbon budgets and their time horizon in Section 3, which presents the model’s numerical application.

There are $m=1,2,...,M$ different *models* linking carbon budgets to future consumption, and we denote this set of models by $\mathcal M$. Conditional on carbon budget *b*, model *m* implies a probability distribution $\pi ^m_t(\cdot |b)$ on relative consumption losses in year *t* compared to a world in which there are no climate damages. Collecting these distributions across models, we define the set^3^

1 $$\begin{aligned} \Pi _b\equiv \{\pi ^m(\cdot |b): \; m \in \mathcal M\}, \end{aligned}$$

summarizing the uncertainty of future consumption losses conditional on carbon budget *b*, as captured by all available models.

**Convex hulls.** In the analysis we pursue, we go beyond set $\Pi _b$ by considering not only the distributions that make it up, but also the set of all their *convex combinations*. That is, for each carbon budget *b* we introduce and focus on the *convex hull* of $\Pi _b$, which we denote by $CH_b$. Formally,

2 $$\begin{aligned} CH_b \equiv \left\{ \sum _{m=1}^{M}\lambda _m \pi ^m(\cdot |b): \; \varvec{\lambda }\ge \varvec{0}, \; \sum _{m=1}^{M}\lambda _m=1\right\} . \end{aligned}$$

We assume that the set $CH_b$ encapsulates the entire universe of uncertain beliefs regarding the effect of carbon budget *b* on future consumption losses. Is this a sensible choice? An oblique way of addressing this question is to imagine examples in which the consideration of convex combinations is problematic. Such examples tend to involve cases in which there is some prior knowledge restricting the range of the “true” distribution. For instance, suppose we wish to make a decision on the basis of our shower’s temperature. There are two experts, one of which claims that the water is freezing and the other that it is boiling hot. Suppose, further, that we *know* that one of the two experts *must* be correct (this may be because our water mixer is broken and unable to modulate between the two extremes). In this case, there is really no point in considering the convex combinations of the experts’ beliefs. It would suffice to simply perform our analysis with these single two distributions and any extra information would simply serve to muddy the water.

Do the socio-economic effects of climate-change policy fall into the above category? We do not see how they could. Probabilistic projections of consumption losses are such that no expert (or model, or set of assumptions) is expected to be exactly “right.” Like most questions in social science, the economic impact of carbon budgets on future consumption patterns cannot be neatly summarized with unique probability distributions, even if the latter are updated over time with Bayesian methods. Instead, it seems reasonable to assume that the truth lies in some middle-of-the-road estimate that splits the difference between the various existing probabilistic models. If we accept this proposition, then it makes sense to consider the convex hull of all probability distributions as defining a probabilistic “realm of the possible” that can be used to guide decision-making.^4^

A satisficing framework. A recurring feature of climate-change negotiations is policymakers’ reluctance to engage with traditional economic analysis. The intertemporal optimization models used by economists are deemed esoteric and overly dependent on assumptions that laymen cannot fully grasp. In addition, the false sense of determinism that a single optimal solution provides may be a source of well-justified suspicion. Still, as alluded to in the Introduction, policymakers seek simplicity. In the context of our paper’s focus on carbon budgets as instruments for climate change policy, we translate this need into the following question:

**Q1:** If carbon budget *b* is chosen, is the probability that future consumption losses are no greater than $L\%$ at least *p*?

In climate negotiations, policymakers tend to gravitate towards this kind of goal-oriented mindset when weighing the relative merits of different policies. Indeed, the much vaunted 2$^{\circ }$C target is an example of a non-optimized goal policymakers seek to meet. It satisfies some requirements on the prevention of major natural disasters, but certainly it is not the result of any conscious optimization effort.

For any given distribution of future consumption losses, we can definitely answer the above question with a simple yes or no. In this case, Q1 is analogous to asking whether the *p-quantile* of the distribution of future consumption losses (conditional on carbon budget *b*) is at least *L*.^5^ Such clarity is impossible in an environment of model uncertainty where multiple distributions of future consumption losses conditional on *b* need to be taken into account. This means that the preceding question must be modified to reflect probabilistic ambiguity. We propose the following adaptation:

**Q2:** If carbon budget *b* is chosen, what proportion of distributions in $CH_b$ keep future consumption losses to at most $L\%$ with probability at least *p*?

The parameters *L* and *p* are real numbers satisfying $L \in [0,100]$ and $p\in [0,1]$.

Put differently, we are interested in the proportion of distributions within $CH_b$ whose *p*-quantiles do not exceed *L*. As we are focusing on proportions, we are implicitly assigning equal weight to all the distributions in $CH_b$. In the parlance of Bayesian statistics, we are assigning a uniform prior on all the distributions of $CH_b$.

Let us now add some formalism to make the above a little more precise. We focus on future consumption losses with respect to a world without any climate change damages. This is clearly a continuous random variable with support [0, 1]. For tractability, we discretize consumption losses in intervals of length 1/*I* where *I* is a natural number. Let $\Delta ^{I-1}$ denote the $(I-1)$-dimensional simplex, i.e., $\Delta ^{I-1}=\{\varvec{\pi }\in \mathfrak {R}^I: \; \varvec{\pi }\ge \varvec{0}, \; \sum _{i=1}^I \pi (i)=1\}$. Given a distribution $\varvec{\pi }\in \Delta ^{I-1}$, let $\pi (i)$ denote the probability of a consumption loss of $i\%$. Then, the set of distributions satisfying the sustainability requirement outlined above is given by the following expression:

3 $$\begin{aligned} \Pi (L,p)=\left\{ \varvec{\pi }\in \mathfrak {R}^I: \; \varvec{\pi }\ge \varvec{0}, \; \sum _{i=1}^I \pi (i)=1,\; \sum _{i \le L}\pi (i) \ge p\right\} . \end{aligned}$$

The intersection of $CH_b$ with $\Pi (L,p)$, denoted by $CH_b(L,p)$, includes all the distributions of $CH_b$ whose *p*-quantiles do not exceed *L*. Formally, it is given by:

4 $$\begin{aligned} CH_b(L,p)\equiv \left\{ \varvec{\pi }\in \mathfrak {R}^I: \; \varvec{\pi }\in CH_b, \; \sum _{i\le L}\pi (i) \ge p\right\} . \end{aligned}$$

With the above definitions and Eqs. (2) and (4) in place, we assume that the performance of a carbon budget *b* is measured by the following ratio:

5 $$\begin{aligned} V_L^p(b)\equiv \frac{{\int \limits _{\mathfrak {R}^I}}\{\varvec{\pi } \in CH_b (L,P)\}{\text {d}} \varvec{\pi }}{\int \limits _{\mathfrak {R}^I}}\equiv \frac{Vol(CH_b(L,p))}{Vol(CH_b)}, \end{aligned}$$

where *Vol* denotes volume in *I*-dimensional space.

Thus, given a carbon budget *b*, the quantity $V_L^p(b)$ calculates the *proportion* of distributions belonging to $CH_b$ that ensure consumption losses of *no more* than $L\%$ with probability *at least* *p*. (Put differently, the quantity $V_L^p(b)$ calculates the proportion of distributions belonging to $CH_b$ whose *p*-quantiles do not exceed *L*). The greater this quantity is the better, for any choice of *L* and *p*.

### Computation

Granting that criterion $V_L^p$ provides a sound basis for comparing alternative carbon budgets, is it computationally tractable? In engaging with this question, it is immediately clear that the high-dimensional integrals in Eq. (5) pose a formidable challenge. The usual way of proceeding is via approximations based on Monte Carlo simulation. This approach, however, can be both computationally costly as well as inaccurate, especially when working in high-dimensional settings such as ours.^6^

We thus take a different approach that draws on results from computational geometry (Bueler et al. [16]). This enables us to efficiently calculate the *exact* value of $V_L^p(b)$, without resorting to any approximations whatsoever. The key trick is to exploit the uncertainty sets’ $CH_b$ and $CH_b(L,p)$ polyhedral structure and reduce the computation of Eq. (5) to a smaller problem, which in turn can be tackled by standard volume-computation algorithms. Essential to this result is the linearity of the constraint in Eq. (3).

To this end, suppose that $I \ge M$, i.e., that the dimension of the consumption space is greater than the number of models. This is an innocuous assumption since consumption losses are a continuous variable, typically discretized in intervals of (arbitrarily) small length (e.g., intervals of 1%), and the number of climate models is generally no more than 10 or 20.^7^ Define the $I \times M$ matrix (the $\pi ^i(\cdot |b)$’s are implicitly assumed to be column vectors)

$$\mathbf \Pi _b\equiv \left[ \pi ^1(\cdot |b), \pi ^2(\cdot |b),...,\pi ^M(\cdot |b)\right]$$

collecting all the distributions in set $\mathcal P_b$. We assume the matrix $\mathbf \Pi _b$ has full column rank, i.e., that the elements of $\Pi _b$ are linearly independent (if this is not the case, we drop one of the linearly dependent distributions at random and continue). Let us define now the linear transformation $T_b: \mathfrak {R}^M \mapsto \mathfrak {R}^I$, where

$$T_b(\varvec{x}) = \mathbf \Pi _b \cdot \varvec{x} = \sum _{m=1}^M \pi ^m(\cdot |b) x_m.$$

Now, consider the sets

$$\begin{aligned} \Lambda= & {} \left\{ \varvec{\lambda }\in \mathfrak {R}^M: \; \varvec{\lambda } \ge \varvec{0}, \sum _{m=1}^M \lambda _m =1\right\} , \\ \Lambda _b(L,p)= & {} \left\{ \varvec{\lambda }\in \mathfrak {R}^M: \; \varvec{\lambda } \in \Lambda , \; \sum _{m=1}^M \lambda _m \left( \sum _{i \le L} \pi ^m(i|b)\right) \ge p\right\} . \end{aligned}$$

Clearly, $CH_b$ and $CH_b(L,p)$ are equal to the images under $T_b$ of $\Lambda$ and $\Lambda _b(L,p)$, respectively.^8^ Since matrix $\mathbf \Pi _b$ is assumed to have full column rank, elementary linear algebra implies:

6 $$\begin{aligned} Vol(CH_b)= & {} \sqrt{\text {det}[\mathbf \Pi _b'\cdot \mathbf \Pi _b]} \; Vol(\Lambda ) \end{aligned}$$

7 $$\begin{aligned} Vol(CH_b(L,p)))= & {} \sqrt{\text {det}[\mathbf \Pi _b'\cdot \mathbf \Pi _b]} \; Vol(\Lambda _b(L,p)). \end{aligned}$$

As a result, Eqs. (6)-(7) yield

8 $$\begin{aligned} V_L^p(b)=\frac{Vol(\Lambda _b(L,p))}{Vol(\Lambda )}.\end{aligned}$$

This is very good news because it means that the problem’s dimensionality has been reduced from *I*, typically a large number, to *M*, the number of different models. Since $\Lambda =\Delta ^{M-1}$, where $\Delta ^{M-1}$ denotes the $(M-1)$-dimensional simplex, we have $Vol(\Lambda )=\frac{\sqrt{M}}{(M-1)!}$. Furthermore, we can use the equality constraints in $\Lambda$ and $\Lambda _b(L,p)$ to eliminate a variable and reduce their dimension to $M-1$. After this elimination, the denominator of Eq. (8) becomes $\frac{1}{(M-1)!}$. Conversely, we can compute the value of the numerator using insights from computational geometry and volume computation (see Bueler et al. [16]). In this paper’s numerical exercise, we use an efficient Matlab implementation of state-of-the-art volume computation algorithms due to Herceg et al. [17].

### Extensions

The power and efficiency of the volume-computation algorithms we employ mean that the decision-making criterion of Eq. (5) can be extended in a number of meaningful directions. In particular, the following enhancements can be made to the basic model of Section 2:

(i) multiple linear (in $\varvec{\pi }$) constraints. For instance, we could add to set $CH_b(L,p)$ a constraint imposing that the expected value of future consumption losses not exceed some limit. Analogously, we could include similar bounds on higher moments of future consumption losses.^9^ In addition, if we had data on the distribution of consumption across and within countries, we could have used them to impose “equity” requirements of various types. As long as the additional constraints are linear in $\varvec{\pi }$, the underlying structure of the problem does not change. We can perform a similar reduction in the problem’s dimensionality as in Eq. (8) and subsequently use the same algorithm as before to calculate volumes.
(ii) *multiple indicators*. For example, staying within the climate-change setting, we could consider not only probability distributions on future consumption but also on pure temperature increase. Setting bounds on the latter could be considered a sort of “ecological” constraint, similar in spirit to the ones considered in the stochastic viability literature (e.g., [19, 20, 23]). Such an operation would increase the problem’s dimensionality considerably, but it can be addressed, so long as the total number of distributions across indicators is not excessive.
(iii) *weighting function on different pdfs.* We could expand the model by introducing a weighting function for the pdfs in $CH_b$ that captures a decision-maker’s confidence in the various models. Suppose we denoted such a weighting function by $f(\cdot )$. Then, the decision criterion of Eq. (5) becomes
  $$V_L^p(b)=\frac{\int \limits _{\mathfrak {R}^I} \varvec{1}\{\varvec{\pi }\in CH_b(L,p)\}\; f(\varvec{\pi }) {\text {d}} \varvec{\pi }}{\int \limits _{\mathfrak {R}^I} \varvec{1}\{\varvec{\pi }\in CH_b\}\;f(\varvec{\pi }) {\text {d}} \varvec{\pi }}.$$
  In contrast to (i) and (ii), this extension to the model poses formidable conceptual and computational challenges. On the conceptual side, there is little consensus on how to come up with a rigorous, theoretically grounded way of explicitly weighting the various climate-economy models and the estimates they produce. Indeed, with regard to the specific climate application in our paper, the choice of mitigation costs (bottom-up or top-down), and especially the functional form of the damage function (quadratic, exponential, or sextic) cannot be adjudicated by current data. This means that there is no clear way we can weight the probabilistic models that are derived from these assumptions. On the technical side, the introduction of a weighting function would significantly complicate the computation of the decision criterion. This is because we can no longer use algorithms of volume computation and would instead need to tackle the computation of multidimensional integrals across convex polytopes –a much harder problem. Along similar lines, it is not clear how we would be able to reduce the problem’s dimensionality from *I* to *M*, where we to stray from the volume computation framework.

### Discussion

The decision-making criterion of Eq. (5) is a particular kind of satisficing criterion adapted to a setting of model uncertainty. The goal that decision-makers want to meet (or, alternatively, the constraint they want to satisfy) is that of ensuring that the *p*-quantile of consumption losses does not exceed a threshold *L*. As such, it is similar to satisficing measures that focus on so-called success probabilities [10–12].^10^ In our setting, a success occurs if the distribution’s *p*-quantile is smaller than *L*. Adapted to an environment with multiple probability distributions, we are interested in the proportion of such “virtuous” probability distributions that a particular carbon budget induces. Ultimately wish to choose a carbon budget that maximizes this proportion. In addition, this criterion can be viewed as an approximate special case of the one proposed and axiomatized by Ahn [13].

How can we actually use the criterion of Eq. (5) in the selection of policy? Answering this question implies the choice of a $L-p$ combination, or at the very least a range of such combinations, on which to apply the $V_L^p$ criterion. Selecting such a $L-p$ combination in a structured, non ad hoc way, is not obvious. If we are lucky, robustness standards of this sort could be dictated by law or custom. In their absence, it becomes incumbent on the decision-maker to develop a way of ordering policies on the basis of their performance vis-a-vis the criterion of Eq. (5) *across a range of plausible L and p*. This is reminiscent of the approach adopted in the Robust Decision-Making literature (Lempert et al. [24]).

A different framework for dealing with probabilistic bounds of the sort explored in this paper can be found in the literature on stochastic programming and chance-constrained optimization (Shapiro et al. [31]). Chance-constrained optimization (CCO) is characterized by optimization problems with linear objectives and constraints that are expressed as probabilistic bounds (Erdogan and Iyengar [32]). Portfolio optimization problems with constraints involving VaR bounds are classic examples of CCO. Despite its intuitive nature, the applicability of CCO has been hindered by the fact that feasible regions of CCO problems will rarely satisfy convexity. For this reason, the literature has largely focused on developing tractable approximations based on constraint sampling and statistical learning techniques. Some progress along these lines has also been achieved in the more challenging case of *ambiguous* CCO, wherein the probabilistic constraints are subject to model uncertainty (Erdogan and Iyengar [32]).

## Application

In this section, we apply the decision-theoretic criterion of Eq. (5) to climate-change data from Drouet et al. [18]. Using the most recent modeling output from the three IPCC AR5 working groups, Drouet et al. developed a novel statistical framework to derive a *set* of probability distributions linking carbon budgets to future damages, consumption, and welfare. These probability distributions are built on the basis of different (but plausible) modeling assumptions regarding (i) mitigation costs, (ii) temperature dynamics, and (iii) climate-related damages. For the purposes of our analysis, we disregard uncertainty in temperature dynamics and retain six of Drouet et al. [18] modeling assumptions corresponding to the different combinations of mitigation costs (top-down and bottom-up) and climate damages (quadratic, exponential, and sextic damage function).^11^ We do so because we find that the latter two factors account for a much greater proportion of the variation in 2100 consumption levels.

In the present paper, we draw from the part of Drouet et al. analysis that connects carbon budgets to consumption losses in year 2100. Consumption losses are defined as (percentage) per-capita consumption reductions relative to a hypothetical baseline in which there is both no climate policy and no climate-related damages. The latter scenario represents an idealized world in which climate change is harmless and no limitations are imposed on emissions. The notion of per capita consumption that we are using is defined in the second page of the Methods section of Drouet et al. [18]. This formulation is standard in the climate-change economics literature.

At this point, one may legitimately call into question the length of the model’s time horizon and the decision to focus on consumption losses so far into the future. Let us address these concerns. Our work, like many other papers in the literature, focuses on year 2100 for two main, interrelated reasons. First, considering the entire twenty-first century is compelling from a policy standpoint, as the Paris climate agreement aims to ”keep a global temperature rise this century well below 2 degrees Celsius above pre-industrial levels.” Second, cumulative emissions until 2100 are considered to be a robust statistical indicator of temperature increase, regardless of the exact trajectory that is taken to arrive at the 2100 cumulative amount (Meinshausen et al. [28], Steinacher et al. [29]).^12^

Indeed, a substantial number of papers that study the socioeconomic effects of climate change use the entire twenty-first century as their time frame and frequently focus on changes in GDP at year 2100. Notable recent examples that provide country-level estimates for per capita GDP losses in year 2100 include Burke, Hsiang and Miguel [33] and Burke, Davis and Diffenbaugh [34]. Other papers that also focus on the entire twenty-first century are Ricke et al. [35], for the computation of country-specific social cost of carbon, and Ueckerdt et al. [36].

Consistent with the range of carbon budgets examined by Drouet et al., we examine nine carbon budgets ranging from 1000 to 5000 GtCO$_2$ in increments of 500. A carbon budget of 1000 GtCO$_2$ represents the adoption of an extremely stringent policy that rapidly accomplishes a complete transition from fossil fuels to renewable energy sources. Conversely, a carbon budget of 5000 GtCO$_2$ represents a business-as-usual energy trajectory that takes no special measures to reduce fossil-fuel use.^13^

We start by focusing on 2100 global consumption losses that are between 5 and 20 percent, i.e., we consider $L \in [5,20]$. Losses in this range are considered very grave, to the extent that they are comparable to major economic calamities such as the Great Recession of 2008 and the Great Depression of the United States in the 1930’s. As such, policy makers should seek to avoid them with high probability, which is why we focus on high values for *p*, namely $p\in [.8,1]$.

**Fig. 1:**
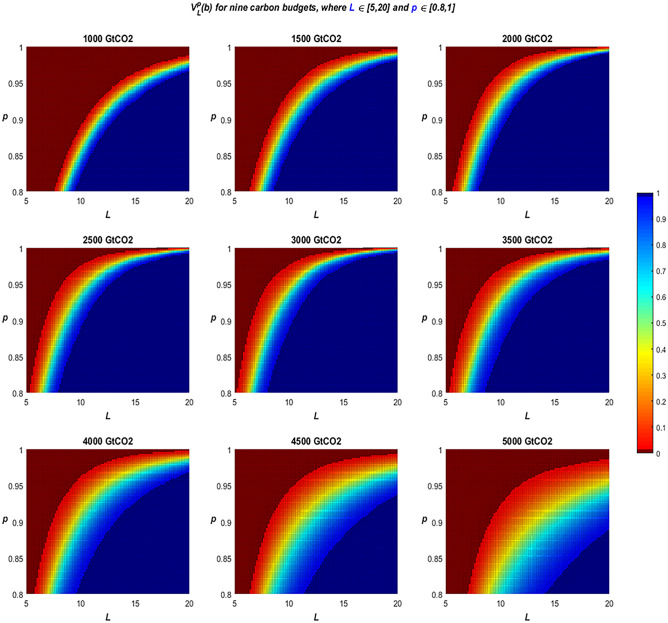
Applying criterion $V_L^p(b)$ to nine carbon budgets using the data of Drouet et al. [18], in the range $L \in [5,20]$ and $p \in [0.8,1]$

Figure 1 summarizes the value of $V_L^p$ for this range of *L* and *p* for the nine carbon budgets under consideration. A clear pattern emerges. High carbon budgets (especially those equaling or exceeding 4000 GtCO2) do uniformly worse for all values of *L* and *p*. The best-performing carbon budget is among the middle-of-the-road choices, ranging from 2000 to 3000 GtCO2.

**Table 1:** $\left[ V_L^p(1000 \text { GtCO}_2),V_L^p(3000 \text { GtCO}_2) ,V_L^p(5000 \text { GtCO}_2)\right]$ evaluated at different levels of *L* and *p* (truncated at two significant digits). A medium carbon budget of 3000 GtCO$_2$ uniformly outperforms its very stringent (1000 GtCO$_2$) and business-as-usual (5000 GtCO$_2$) counterparts. Moreover, business-as-usual is by far the worst option

$\varvec{L\setminus p}$	.80	.85	.90	.95	.99
5	[0, .01, 0]	[0, 0, 0]	[0, 0, 0]	[0, 0, 0]	[0, 0, 0]
10	[1,1,.63]	[.66, 1, .32]	[0, .96, .07]	[0, .29, 0]	[0, 0, 0]
15	[1, 1, 1]	[1, 1, .91]	[1,1,.58]	[.32, 1, .12]	[0, .28, 0]
20	[1, 1, 1]	[1, 1, 1]	[1, 1, .96]	[1, 1, .46]	[0, 1, 0]
25	[1, 1, 1]	[1, 1, 1]	[1, 1, 1]	[1, 1, .82]	[.45, 1, .03]

Table 1 provides additional evidence of this finding. It compares the performance of three carbon budgets (1000-3000-5000 GtCO$_2$), representing stringent, “medium,” and business-as-usual climate policies, across a range of *L* and *p*. It becomes clear that a medium carbon budget uniformly outperforms the two extremes, occasionally significantly so. In fact, for all the $L-p$ combinations appearing in Table 1, it is the *highest-performing* carbon budget among the nine examined (oftentimes uniquely so). This is because its middle-of-the-road approach guards against consumption losses that are due both to high mitigation costs and high climate damages. The differences can occasionally be striking: consider for instance $L=10$ and $p=.9$. Here, a medium carbon budget does exceedingly well, as 96% of all pdfs in $CH_{3000}$ manage to contain losses at 10% with probability at least .9. The corresponding figures for the very stringent and business-as-usual policies are 0 and 7%, respectively. Finally, it should be mentioned, even though it does not appear in Table, that a carbon budget of 2500 GtCO$_2$ is always at least the second-best choice after 3000 GtCO$_2$ (occasionally tying for first), for these combinations of *L* and *p*.

Next, we investigate these nine carbon budgets’ potential of meeting stronger guarantees on consumption losses. In particular, we zero in on losses ranging from 1 to 5 percent. Containing losses to such modest levels would represent a very good outcome for the world. Yet, current estimates suggest it may be too late to attain, at least with a reasonable degree of confidence.

**Fig. 2:**
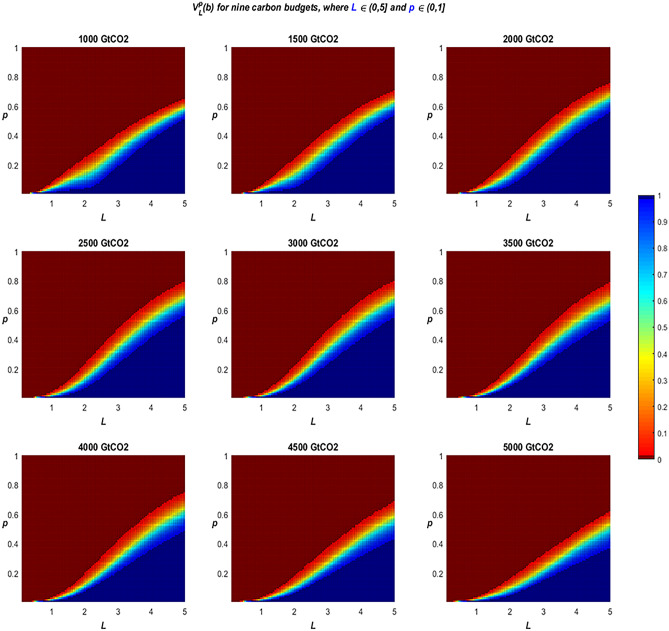
Applying criterion $V_L^p(b)$ to nine carbon budgets using the data of Drouet et al. [18], in the range $L \in [1,5]$ and $p \in (0,1]$

Figure 2 depicts the relevant results, and Table 2 summarizes a set of corresponding $V_L^p$ values for the same three carbon budgets (very stringent, medium, and business-as-usual) mentioned before. The patterns previously observed in Fig. 1 are still present in Fig. 2. It is evident that middle-of-the-road carbon budgets (2000-3000 GtCO2) offer the best chance of containing consumption losses to modest levels. The only exception to this statement applies to very low damages. For example, in Table 2 we see that a little more than a fifth of the pdfs in $CH_{b}$ for $b=1000$ GTCO$_2$ imply losses of $L\le 1$ with probability at least .05, whereas no other carbon budget achieves losses this low with probability at least .05. That said, $p = .05$ is a low probability offering little insurance against such losses, so it would be wise not to make too much of this fact.

**Table 2:** $\left[ V_L^p(1000 \text { GtCO}_2),V_L^p(3000 \text { GtCO}_2) ,V_L^p(5000 \text { GtCO}_2)\right]$ evaluated at different levels of *L* and *p* (truncated at two significant digits)

$\varvec{L\setminus p}$	.05	.10	.20	.40	.60	.80
1	[.21, 0, 0]	[0, 0, 0]	[0, 0, 0]	[0,0,0]	[0, 0, 0]	[0, 0, 0]
2	[.95, 1, .92]	[.72, .80, .32]	[.10, .03, 0]	[0, 0, 0]	[0, 0, 0]	[0, 0, 0]
3	[1, 1, 1]	[1, 1, 1]	[.95, 1, .59]	[.02, .10, 0]	[0, 0, 0]	[0, 0, 0]
4	[1, 1, 1]	[1, 1, 1]	[1, 1, 1]	[.87, .98, .25]	[0, .10, 0]	[0, 0, 0]
5	[1, 1, 1]	[1, 1, 1]	[1, 1, 1]	[1, 1, .93]	[.22, .87, .03]]	[0, .01, 0]

Relation to expected maxmin utility. A prominent decision-theoretic framework for choice under model uncertainty is that of maxmin expected utility (Gilboa and Schmeidler [37]). According to this criterion, a policy is preferred to another if and only if it has higher minimum expected utility (across the set of possible distributions). In the context of our application, a carbon budget is preferred to another if and only it has lower maximum expected consumption losses in year 2100 across the six pdfs of Drouet et al. [18].

**Table 3:** $EL_i(b)$ denotes expected consumption losses in year 2100 given carbon budget *b* and pdf *i* of Drouet et al. [18]. Pdfs 1-2-3 (resp., 4-5-6) reflect top-down (resp., bottom-up) mitigation costs. Pdfs 1-4 reflect quadratic, pdfs 2-5 exponential, and pdfs 3-6 sextic damages. For each carbon budget, maximum expected damages are indicated in bold

*b* (GtCO$_2$)	$EL_1(b)$	$EL_2(b)$	$EL_3(b)$	$EL_4(b)$	$EL_5(b)$	$EL_6(b)$	$\max _i EL_i(b)$
1000	$0.0732$	0.0662	0.0715	0.0504	0.0444	0.0485	0.0732
1500	$0.0664$	0.0599	0.0661	0.0435	0.0367	0.0435	0.0664
2000	0.0612	0.0540	$0.0631$	0.0392	0.0322	0.0415	0.0631
2500	0.0578	0.0505	$0.0638$	0.0369	0.0302	0.0426	0.0638
3000	0.0540	0.0485	$0.0655$	0.0362	0.0304	0.0470	0.0655
3500	0.0526	0.0473	$0.0716$	0.0367	0.0316	0.0563	0.0716
4000	0.0508	0.0476	$0.0812$	0.0390	0.0353	0.0699	0.0812
4500	0.0517	0.0510	$0.0970$	0.0428	0.0419	0.0878	0.0970
5000	0.0529	0.0571	$0.1202$	0.0467	0.0515	0.1162	0.1202

Table 3 lists *expected* consumption losses in year 2100 across all combinations of carbon budgets and probability distributions of Drouet et al [18]. Maximum expected consumption losses (across the six pdfs of Drouet et al) for each carbon budget are denoted in Table’s last column. The carbon budget that minimizes such maximum expected losses is 2000 GtCO$_2$, with 2500 GtCO$_2$ and 3000 GtCO$_2$ closely behind it. In all three cases, the pdf which maximizes consumption losses is the one corresponding to sextic damages and top-down abatement costs. We conclude that the minmax expected loss criterion yields results that are broadly in line with those of our framework: middle-of-the-road carbon budgets, lying within the 2000-3000 GtCO$_2$ range, do better than their more extreme counterparts. As the minmax criterion leads to a complete ordering of carbon budgets, we are also able to say that among those medium carbon budgets 2000 GtCO$_2$ does slightly better than 2500 GtCO$_2$, which in turn does slightly better than 3000 GtCO$_2$.

### Comments

It is worth briefly reiterating the distinguishing features of our approach with respect to the rest of the literature. First of all, by focusing squarely on bounding future consumption losses, we adopt a satisficing, as opposed to optimizing, analytic framework. This allows us to avoid all the attendant controversy of the optimization setting regarding the selection and justification of discount rates, rates of intertemporal substitution, coefficients of risk aversion, objective functional forms, and so on. Instead of solving a complex problem of intertemporal optimization, our primary goal is to assess the potential of different policies to stave off heavy consumption losses. This is, in a sense, similar to focusing on the goal of avoiding worst-case future outcomes without caring about how that task is accomplished across time. The suitability of such a modeling framework is debatable, but at the very least it offers a fresh perspective on the assessment of environmental policy.

Second, we explicitly take into account model uncertainty regarding the damages higher temperatures will cause to economic activity, and the costs of climate-change mitigation. This means that we can consider many plausible modeling choices simultaneously while remaining agnostic on their relative likelihood. By taking this multiplicity of models into account in a systematic way, we are able to obtain a more robust idea of whether a given carbon budget will be able to keep consumption losses within an acceptable threshold.

**Fig. 3:**
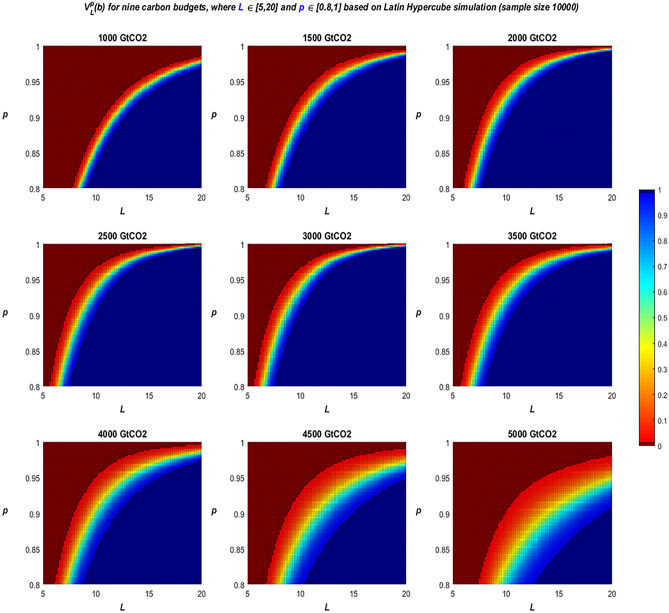
Monte Carlo simulation estimates (using Latin Hypercube sampling) of $V_L^p$ for nine carbon budgets using the data of Drouet et al. [18], in the range $L \in [5,20]$ and $p \in [0.8,1]$. Compared to Figure 1, some of the true uncertainty has been suppressed, with a greater proportion of 0-1 values appearing in the graphs

The combination of the above two elements in a way that does not rely on simulation is novel to the literature, as discussed in the Introduction. With regard to the actual results that our framework yields in the numerical example, we want to stress that caution is in order. This is because these results could plausibly change, where we have to consider additional modeling choices leading to a broader set of pdfs linking carbon budgets to economic outcomes and/or adopt an altogether different, though equally defensible, long-term goal. Instead, we view the primary importance of the numerical exercise as a proof of concept for the analytic framework as laid out in Section 2. Furthermore, it is worth reiterating that results from computational geometry allow us to obtain exact results for the value of the criterion of Eq. (5), without resorting to Monte Carlo simulations of uncertain accuracy. Along these lines, in Fig. 3 of section A1 of the Appendix we show how Monte Carlo simulation with Latin-hypercube sampling may sometimes provide incorrect estimates.

## Conclusion

This paper has presented a model for decision-making under model uncertainty. Its main conceptual departure from existing work is the integration of ideas from the literature on satisficing (Simon [6, 8]) into an ambiguity-aversion framework. The decision criterion that we propose is an adaptation of the success-probability criterion (Castagnoli and LiCalzi [11]) to a setting of non-unique probability distributions linking actions to consequences. The integration of the model-uncertainty and satisficing literatures in a non-simulation-based framework is novel^14^, as is the application of results from computational geometry to obtain precise results. On the latter front, these computational techniques mean that we do not have to use Monte Carlo simulations of dubious accuracy to approximate our results.

We apply our decision criterion to a set of distributions derived by Drouet et al. [18] linking carbon budgets to future consumption losses. The main finding of this numerical study is the superior performance of medium carbon budgets in preventing grave consumption losses with high probability. These results, however, should be taken with a grain of salt, and we caution against drawing overconfident policy implications. Instead, we view the main contribution of the empirical exercise as a proof of concept for the proposed theoretical framework.

Along similar lines, it is worth emphasizing that focusing only on the effects of climate change in year 2100 introduces important limitations to the present study. We highlight two which are particularly prominent. First, ignoring emissions dynamics means that we cannot comment explicitly on the intertemporal tradeoffs that are inherent in climate policy. For example, an ambitious carbon budget might imply emission cuts for the current generations that are politically infeasible or borderline intractable from a technological standpoint. Such information would be worth taking into account in a systematic way as we assess the desirability of different carbon budgets on the basis of their effects on year 2100. To some extent, these kinds of intertemporal considerations are already captured by the pdfs of Drouet et al. [18] but a clearer picture would be very useful.

Second, by disregarding the exact way in which we arrive at 2100 cumulative emissions targets, our model is not acknowledging that trajectories with similar carbon budgets may imply different levels of climate risk. This is due to the fact that climate dynamics are nonlinear and potentially rife with tipping points and dangerous thresholds that, once exceeded, can provoke runaway climate change and irreversible damage (e.g., shutdown of the thermohaline circulation, permafrost melt). In the setting of our model, there might be more than one way of achieving a middle-of-the-road carbon budget, with a significant portion of them being quite dangerous. When one takes such information into account, it may suggest that a lower carbon budget should be chosen, even though the decision criterion of the model may say otherwise. Once again, the pdfs of Drouet et al. [18] do implicitly take these risks into account, but a more transparent treatment would be desirable.

Fruitful avenues for future research would seek to enhance both the theory as well as the applied section of the paper. On the theoretical side, it would be interesting to develop a way of ordering policies on the basis of their performance vis-a-vis the criterion of Eq. (5) *across a range of L and p*. It is important to consider such ranges because it may often be hard to justify the choice of a unique specific $L-p$ combination to apply the criterion to. Here, methods from the social-choice literature on voting (see, e.g., [38] for an application regarding indices of multidimensional welfare) could be useful. On the applied side, follow-up work could seek to improve and enrich the set of probability distributions linking carbon budgets to future economic and social conditions. Along these lines, various tipping points (relating to, e.g., permafrost melt, ecosystem collapse, thresholds of tropical deforestation, etc) could be better taken into account. Finally, it would be interesting to apply the satisficing framework to alternative long-term policy goals that go beyond bounding consumption losses.

## Data Availability

Availability of data and material (data transparency): excel file with distributions

## Appendix

### Comparison with Monte Carlo Simulation Based on Latin Hypercube Sampling

It is reasonable to ask how our geometric technique compares to the results of an equivalent simulation exercise. To answer this question, we performed the exact same computations by using Latin Hypercube sampling to sample 10000 points in the five-dimensional simplex. This leads to roughly similar running time. Fig. 3 summarizes the relevant results. Comparing it to Fig. 1, we notice qualitatively similar patterns regarding the superior performance of medium carbon budgets and poor performance of business-as-usual scenarios. However, we also see that the simulation-based method does not fully capture the true range of the $V_L^p$ criterion, as it tends to expand the area of the $L-p$ graphs with binary 0-1 values. This imprecision is relatively harmless in the current example but could become problematic when there is greater divergence between the pdfs whose convex hull we are considering. Higher dimensionality could also pose significant hurdles for a pure simulation-based approach due to the “curse of dimensionality.” 

## Footnotes

1 “Evidently, organisms adapt well enough to *satisfice*; they do not, in general, *optimize*...a satisficing path [is] a path that will permit satisfaction at some specified level of all its needs.” Simon [7]. We thank a referee for bringing this quote to our attention.

2 Another good example of this link between classical satisficing and viability is Karacaoglu et al. [39].

3 Since the analysis will concentrate on year 2100, in what follows we omit the subscript *t*.

4 Note that such polyhedral uncertainty sets are often encountered in the decision-theoretic literature (e.g., Olszewski [14], Danan et al. [15]) and its applications to environmental policy (Athanassoglou and Bosetti [40], Danan et al. [15]).

5 It is worth noting the extremely close relationship of loss quantiles to Value-At-Risk (*VaR*), a risk measure that is ubiquitous in finance. Given a random variable *X* of losses related to a risky position, $VaR_{\alpha }(X)$ is equal to the $1-\alpha$-quantile of *X*.

6 Any meaningful discretization of consumption losses—a continuous variable—will be high-dimensional.

7 If for some reason we wanted to impose $M >I$ (so that our problem is effectively already low-dimensional), we would proceed directly without reducing Eq. (5) to Eq. (8).

8 Note how $$\sum _{i \le L} \left( \sum _{m=1}^M \lambda _m \pi ^m(i|b)\right) = \sum _{m=1}^M \lambda _m \left( \sum _{i \le L} \pi ^m(i|b)\right)$$.

9 Note that moment constraints can be made linear by raising both sides of the inequality to the corresponding inverse power.

10 Its consideration of quantiles is also somewhat reminiscent of the quantile-maximization model of Rostek [41].

11 Specifically, looking at Section S12 of Drouet et al. supplementary information, we assume temperature is fixed to “climate-all” and consider all combinations of {mitigation-BU, mitigation-TD} and {damage sextic, damage quadratic, damage exponential}.

12 To be clear, the cited studies find that (distinct) emissions trajectories that have the same cumulative emissions in year 2100 tend to imply similar levels of warming for year 2100.

13 By way of comparison, cumulative emissions from 1850 up to and including 2017 stood at roughly 1540 GtCO$_2$ (see https://www.globalcarbonproject.org/carbonbudget/18/highlights.htm). A graph of the entire trajectory of global cumulative emissions, including the relative contributions of different regions, can be found at https://ourworldindata.org/grapher/cumulative-co2-emissions-region?stackMode=absolute.

14 Though we remind the reader that our model can be viewed as a special case of the more general framework of Ahn [13].
